# EcoSpec: Highly Equipped Tower-Based Hyperspectral and Thermal Infrared Automatic Remote Sensing System for Investigating Plant Responses to Environmental Changes

**DOI:** 10.3390/s20195463

**Published:** 2020-09-23

**Authors:** Yuki Hamada, David Cook, Donald Bales

**Affiliations:** Argonne National Laboratory, Lemont, IL 60439, USA; dsgcook@prodigy.net (D.C.); donaldbales@icloud.com (D.B.)

**Keywords:** hyperspectral remote sensing, photosynthesis, ecosystem functions, spectral reflectance, near surface, vegetation indices, photosynthetically active radiation, climate change, multiple scale, agriculture, crop monitoring

## Abstract

Despite an advanced ability to forecast ecosystem functions and climate at regional and global scales, little is known about relationships between local variations in water and carbon fluxes and large-scale phenomena. To enable data collection of local-scale ecosystem functions to support such investigations, we developed the EcoSpec system, a highly equipped remote sensing system that houses a hyperspectral radiometer (350–2500 nm) and five optical and infrared sensors in a compact tower. Its custom software controls the sequence and timing of movement of the sensors and system components and collects measurements at 12 locations around the tower. The data collected using the system was processed to remove sun-angle effects, and spectral vegetation indices computed from the data (i.e., the Normalized Difference Vegetation Index (NDVI), Normalized Difference Water Index (NDWI), Photochemical Reflectance Index (PRI), and Moisture Stress Index (MSI)) were compared with the fraction of photochemically active radiation (fPAR) and canopy temperature. The results showed that the NDVI, NDWI, and PRI were strongly correlated with fPAR; the MSI was correlated with canopy temperature at the diurnal scale. These correlations suggest that this type of near-surface remote sensing system would complement existing observatories to validate satellite remote sensing observations and link local and large-scale phenomena to improve our ability to forecast ecosystem functions and climate. The system is also relevant for precision agriculture to study crop growth, detect disease and pests, and compare traits of cultivars.

## 1. Introduction

Atmosphere, plants, and soils control terrestrial carbon and water cycles [1,2,3,4,5]. Better understanding of ecosystem heterogeneity and dynamics at the biosphere–atmosphere interface is needed for accurately forecasting future climate and contributions and responses of the terrestrial biosphere [1,4]. While our ability to forecast ecosystem functions and climate at regional and global scales has significantly advanced, little is known about how local phenomena such as heterogeneity in water and carbon fluxes at a daily time scale relate to large-scale phenomena and vice versa [4,6]. Understanding ecosystem functions and climate change interactions is a critical knowledge gap, and such interactions need to be better represented in climate change models [4,6,7,8,9]. Researchers have found strong temporal correlations between meteorological variables (e.g., temperature and photosynthetically active radiation) and ecosystem properties (e.g., photosynthesis and respiration) at landscape and regional scales or at low temporal frequency [7,8,9,10]. Near-surface remote sensing would play an important role in experimenting across spatial and temporal scales and filling the knowledge gap [11].

Gaining insights for local-scale ecosystem functions using near-surface remote sensing is not a new concept. Spectroscopy, fluorescence sensing, and thermal imaging are three examples of the most promising near-surface remote sensing techniques for studying plant responses to environmental conditions at a fine scale [12]. Plant spectroscopy employs observations of spectral reflectance of plants across a contiguous spectral region that are often collected in the field or laboratory using a spectrometer. Such detailed measurements of plant spectral reflectance are known to provide information about plant traits that play an important role in plant photosynthesis [13] and other functions through establishing empirical relationships between their traits and spectral reflectance. Thus, plant spectroscopy provides a noninvasive, nondestructive alternative to traditional methods for collecting plant trait measurements. Sakowaska et al. [14] developed a tower-based hyperspectral system, WhiteRef, to automatically collect spectral reflectance measurements at a Mediterranean grassland. Spectral vegetation indices derived from the hyperspectral reflectance measurements collected by the system showed strong correlations with plant pigment and the fraction of photochemically active radiation (fPAR).

Sun-induced chlorophyll fluorescence (SIF) is known to capture rapid change in photosynthesis, and its study is a fast-growing research area in remote sensing of vegetation [15]. To track SIF changes and their correlation with gross primary production (GPP), Yang, et al. [16] developed a tower-based fluorescence measurement system using a spectrometer having a very fine spectral resolution (approximately 0.13 nm) for the 680–775 nm spectral range. The study conducted in temperate deciduous forests showed strong diurnal and seasonal correlations between SIF and GPP estimated using the eddy covariance method (*R*^2^ = 0.82 and 0.73, respectively) as well as with fPAR (*R*^2^ = 0.90 and 0.80, respectively) [16]. Yang et al. [17] further improved the system by optimizing tradeoff between spectral range and resolution. Grossmann et al. [18] integrated a visible band (400–900 nm) into a SIF sensing system to allow for calculating vegetation indices (PhotoSpec). The initial testing based on broad leaves, grass, and dark light showed the stability and robustness of the system for continuously collecting SIF measurements throughout the day [18].

Increased canopy temperature caused by plant water stress is widely studied using thermal infrared (TIR) remote sensing [19,20]. For proximal sensing, Mangus et al. [20] assembled a TIR imaging system using an off-the-shelf TIR camera to evaluate the effectiveness of high spatial resolution canopy temperature in relation to soil moisture to study plant moisture content. Their 80-day study verified the canopy temperature measurement at an accuracy of ±0.62 °C, and the model developed using the TIR measurements revealed strong correlation between the canopy temperature and plant water use [20].

These near-surface remote sensing systems have demonstrated that continuous optical and infrared measurements at atmosphere–biosphere interfaces would fill the data and knowledge gaps toward understanding the linkage between local- and regional/global-scale phenomena by providing new datasets at fine spatial and temporal scales. If we could integrate these sensing technologies and complementary sensors into a single synchronized system, that would enable a range of simultaneous environmental and ecological measurements and allow for various experiments and modeling of local atmosphere–biosphere interactions. This paper presents a highly equipped hyperspectral remote sensing system, the EcoSpec system, that integrates complementary environmental sensors to simultaneously collect measurements with hyperspectral reflectance of land surfaces. The ultimate goal of this system is to enable investigation of temporal patterns and associations between continuous measurements of hyperspectral reflectance of land surface and continuous meteorological and biological measurements under natural and managed environments (e.g., agricultural fields).

## 2. Materials and Methods

### 2.1. Hardware and Physical Architecture

**Figure 1:**
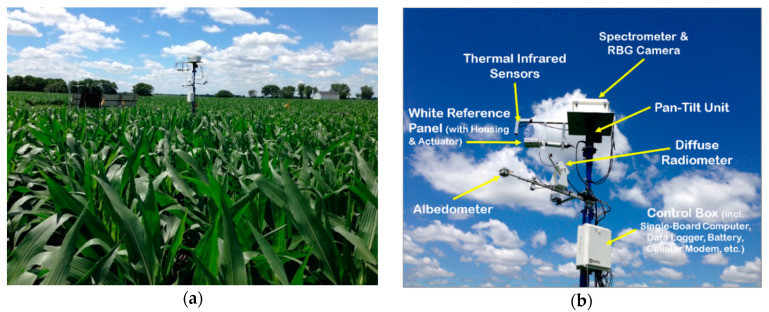
EcoSpec system. (**a**) The system installed in a corn field (2016) powered by solar energy and (**b**) system components.

The EcoSpec system is equipped with five types of optical and infrared sensors powered by solar energy (Figure 1, Table 1, Supplementary Materials, Video S1) installed at two locations on the tower, called the upper and lower packages, respectively.

#### 2.1.1. Upper Package

The upper package consists of a spectrometer and true-color (or red, green, and blue (RGB)) camera, two TIR sensors, a white reference panel with a custom panel housing, and an actuator. All of these components are mounted on a pan-tilt unit (PTU) (Figure 1, Table 1, Video S1). The spectrometer and RGB camera are housed in an enclosure.

The spectrometer (ASD FieldSpec 4; Cambridge, MA, USA) measures electromagnetic radiation (EMR) that is reflected by land surfaces, such as plants and soils (Figure 1). EMR reflected by the land surface is collected across 2151 narrow contiguous spectral channels from a 350 to 2500 nm spectra range with 4 to 10 nm channel width (Table 1). The instrument uses a 1.5 m fiber optic cable having a 25 degree field of view (FOV). The spectrometer is housed in a securely sealed enclosure (ENC 14/16-MM-NC, Campbell Scientific Inc.; Logan, KY, USA) to be protected from elements. The optical fiber cable that is extended from the spectrometer is housed in a metal casing, and the tip of the cable is installed perpendicular to the ground to collect nadir-viewing measurement. The enclosure is placed on a steel plate secured to the top of a pan-tilt platform (PTU-D300, FLIR; Wilsonville, AL, USA) and mounted on the tower.

The RGB camera (Axis Q1604; Lund, Sweden) housed in the enclosure is installed perpendicular to the land surface (Figure 1, Table 1). The camera captures true-color photos from the land surface around the tower via a small glass opening fabricated at the bottom of the enclosure. Because of the large FOV of the camera, each photo contains the FOV of the spectrometer and its vicinity. The RFB photos provide contextual information, such as soil exposure, plant condition, canopy closure, and shadowing, which aid in interpreting the spectrometer and TIR measurements. For example, RGB photos can be used to identify the cause of sudden changes in spectral reflectance and/or vegetation indices and flag those data. The photos may be useful for investigating the effects of soil exposure, leaf orientation, and shadow on optical and infrared measurements.

Two TIR sensors (Apogee Instruments SI-111; Logan, KY, USA), downward-looking and upward-looking, are installed on the side of the metal casing of the fiber cable (Figure 1, Table 1). The downward-looking TIR sensor measures the radiant temperature of land surface consisting of plant canopies. This TIR sensor has approximately 22 degree FOV. The upward-looking sensor measures sky temperature that is indicative of cloudiness of the sky at a given time. Both sensors are connected to a data logger, and readings are recorded over the predefined time periods: 1 and 30 min, respectively.

We developed a reference subsystem using the white reference panel (Labsphere spectralon target SRT-99-050; North Sutton, NH, USA), actuator (Progressive Automation PA 15-8-11; Arlington, TX, USA), and custom housing (Figure 1, Table 1). The 99% reflective Spectralon^®^ panel extends only when reference data collection is needed. Movement of the reference subsystem is synchronized with movement of the PTU and the spectrometer’s preparatory activities, such as parameter optimization (Video S1). The reference subsystem supports reliable optical and infrared reflectance data collection using a spectrometer by collecting white reference data and information on the sensor’s internal noise more frequently than traditional settings (e.g., every 20–30 min)**.**

#### 2.1.2. Lower Package

The lower package consists of an albedometer and diffuse radiometer (Figure 1, Table 1). The albedometer (Schenk Dual-Pyranometer 8104; Grass Valley, CA, USA) measures net broadband radiation and thereby determines how much incoming radiation is absorbed and reflected. Radiation between 300 and 700 nm is mostly absorbed by plants, and these measurements provide the total canopy reflectance across all wavelengths. Downwelling solar radiation can also be compared to top-of-the-atmosphere solar radiation to determine the opacity of the atmosphere, which could provide insights for the amount of diffuse solar radiation.

The diffuse radiometer (Irradiance Rotating Shadowband Radiometer (RSR2); Cambridge, MA, USA) measures direct and diffused light separately and allows for quantifying the incoming radiation components. At least every 30 s, a narrow arm passes over the sensor to cast a shadow. This movement briefly blocks direct sunlight, and the sensor only measures diffused light during that time. The radiometer measures diffused light more frequently under partly cloudy conditions than sunny conditions in order to track rapidly changing irradiance.

The lower package also includes the control box. The control box houses a single-board computer, data logger, small battery, and cellular modem (Figure 1, Table 1). The single-board computer (Raspberry Pi) controls activities of the sensors and components in a synchronized manner, monitors functionality of the upper package, temporally stores measurements, and wirelessly transmits the temporally stored data to the remote server through cellular connection. The data logger (CR1000) logs and records measurements collected using the thermal IR sensors, albedometer, and diffuse radiometer as an average over two time periods: every 1 min and 30 min.

### 2.2. Software, Operation, and Data Collection and Management Design

We developed custom software to operate the EcoSpec system in a Linux environment, including collection of hyperspectral EMR measurements. The software package allows a range of autonomous functions for the system, which include (1) initiating the system 20 min before sunrise each day, (2) triggering activities of the components in a synchronized manner, (3) monitoring system functions and sending an email alert when malfunction is detected, (4) rebooting the system when malfunction is detected, (5) transmitting the data approximately every 30 min throughout the day, and (6) shutting down the system at sunset each day.

The system automatically initiates 20 min before sunrise each day based on the internal clock with the longitude setting. The PTU rotates 300 degrees (1 full rotation of the system) to test the functionality of the platform and return to position 1. The first spectrometer data collection begins at sunrise at position 1. The actuator extends and places the white reference panel under the fiber optic cable (Video S1). The spectrometer optimizes the parameter setting. Once optimized, the spectrometer collects over 100 white reference measurements from the 99% reflective Spectralon^®^ panel and records the average value. The actuator retracts and stores the white panel in the custom housing (Video S1). This protects the surface from elements (e.g., dust) that may impact the integrity of spectral reference measurements. While retracting, the spectrometer measures and records dark current or the sensor’s internal noise. Shortly after, the spectrometer collects measurements from the land surface approximately 500 times and records the average value. A RGB photo of the spectrometer FOV and its vicinity is captured during the reflectance measurements from the land surface. Once it completes a full sequence at its current position, the PTU moves to the next position and repeats the series of steps from the spectrometer optimization to land surface measurements before moving on to a subsequent position (Video S1). While rotating 300 degrees for each rotation, the upper package of the system pauses at 12 positions around the tower and collects measurements from the land surface in order to account for local variability within the study site. The data collection sequence continues throughout the day. At sunset, the system moves to position 1 and automatically shuts down to conserve energy for the next day.

At each position, a set of hyperspectral EMR measurements is comprised of gain, offset, integration time, white reference (2150 features), dark current (2150 features), and land surface measurements (2150 features), and all measurements are temporally recorded in the Raspberry Pi along with measurement identification, PTU position, and sample date and time (Video S1). A set of measurements at a single position typically takes around 1 min. It will take longer when ambient light is low (e.g., cloudy sky conditions, in the early or late day). On a sunny summer day, the system could collect measurements from 800+ data points.

The system allows us to collect an array of land surface measurements, including hyperspectral reflectance of plants and soils, true-color photos capturing contextual information of land surface, radiant temperature of sky and land, incoming and reflected shortwave radiation, and incoming radiation components (direct and diffuse), as frequently as every minute. Several of the sensors are mounted on a pan-tilt unit on top of the tower to collect hyperspectral reflectance, land surface photos, and radiant temperature of sky and land from 12 discrete positions around the tower. This allows for capturing inherent heterogeneity of the terrestrial ecosystem at a local scale and to obtain representative spectral signatures of the plant and soil surfaces via averaging those measurements.

### 2.3. Data Preparation

Hyperspectral reflectance measurements that are collected using the EcoSpec system must be preprocessed prior to analysis. For this paper, we prepared the data using parsimonious processing methods to remove outliers and mitigate sun-angle effects to demonstrate the usefulness of measurements collected using the system.

We first identified outliers in hyperspectral reflectance measurements by applying two criteria that define the allowable range for instantaneous data values (i.e., values greater than 0 and smaller than 0.8) and the allowable change in reflectance values within a limited time period (e.g., absolute change greater than 30% within 30 min). We removed data points that were identified as outliers from the dataset for subsequent processing and analyses. We developed a prototype methodology for minimizing the sun-angle effects on hyperspectral reflectance collected from land surface, known as a bidirectional reflectance distribution function (BRDF). We modeled an overall temporal trend and pattern in reflectance values on each date. Sunny dates had stronger, more symmetric pattern than cloudy dates. We removed the modeled trend and pattern from the reflectance values prior to analyses. To preserve the integrity of the measurements, an independent model was developed for each of the 2150 spectral channels.

After the measurements were preprocessed as described above, we calculated the average hyperspectral reflectance values over 30 min to match the temporal frequency to that of the meteorological data collected using an eddy covariance method. Using the half-hourly hyperspectral data, we computed the Normalized Difference Vegetation Index (NDVI) [21,22], Normalized Difference Water Index (NDWI) [23], the Photochemical Reflectance Index (PRI) [24], and Moisture Stress Index (MSI) [25]. We compared the index values with selected flux tower variables during the season to demonstrate the usefulness of the hyperspectral reflectance measurements collected using the EcoSpec system.

## 3. Results

**Figure 2:**
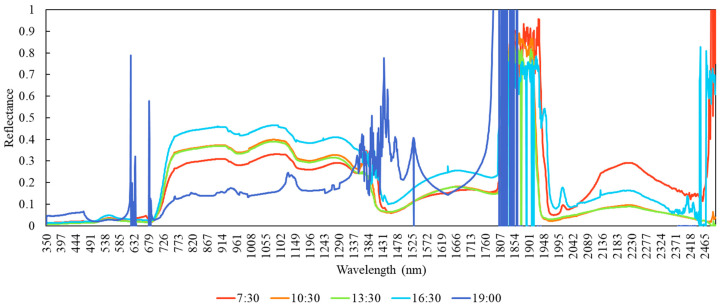
Land surface reflectance from the visible to shortwave infrared spectral region at 7:30 a.m., 10:30 a.m., 1:30 p.m., 4:30 p.m., and 7:00 p.m. at local time on 21 August 2015 (DoY 234).

The following results are examples from the 2015 study conducted at the soybean field in AmeriFlux US-B1 site located within Fermi National Accelerator Laboratory, Batavia, Illinois. Examples of spectral reflectance values of land surfaces that were collected using the EcoSpec system are shown in Figure 2. The spectral reflectance signatures presented in the figure were collected at 7:30 a.m., 10:30 a.m., 1:30 p.m., 4:30 p.m., and 7:00 p.m. at local time on 21 August 2015 (DoY 234). There are two atmospheric absorption features around the 1350–1430 and 1800–1940 nm spectral ranges that are seen across all the time periods. While spectral reflectance signatures show characteristic profiles for healthy vegetation most of the day, the signature appears noisy, and a vegetation spectral signature becomes obscured late in the day (Figure 2). This type of system could help determine the effective daytime period or sun angles and the minimum irradiance to usefully collect optical and infrared information to study plant properties and functions.

**Figure 3:**
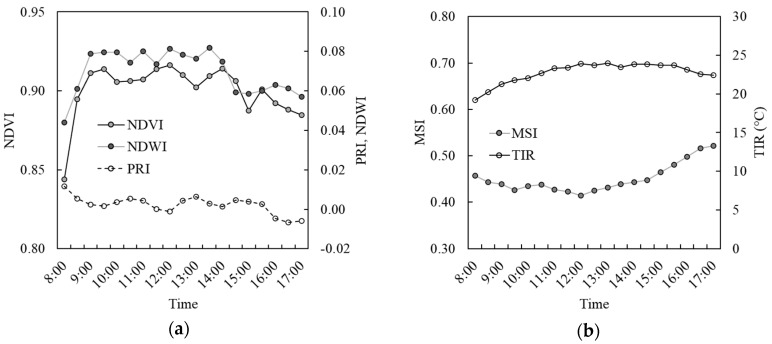
Diurnal trajectories of (**a**) NDVI, NDWI, and PRI calculated from the hyperspectral reflectance measurements and (**b**) the MSI and plant canopy temperature measured by the thermal infrared (TIR) sensor on 21 August 2015 (DoY 234).

Diurnal trajectories of selected spectral vegetation indices and TIR on 21 August 2015 (DoY 234) are shown in Figure 3. The NDVI and NDWI, which are known to correlate with green biomass [21,22,26,27] and plant moisture [23,28,29,30], respectively, follow a similar pattern throughout the day, while the PRI, which is known to indicate photosynthetic radiation use efficiency of plants, shows an opposite pattern (Figure 3a). The MSI, which is known to indicate plant’s moisture stress [25,31,32,33], and TIR both show gradual change during the day (Figure 3b). Plant canopy temperature represented by TIR increases from morning to mid-day and slightly decreases later in the day. On the other hand, plant water stress depicted by the MSI decreases from morning to mid-day and steadily increases toward the evening. This contrasting pattern between the MSI and TIR shows the indication of TIR measurements for plant moisture content or their responses to water availability, which has been demonstrated in a number of studies [19,20,34].

**Figure 4:**
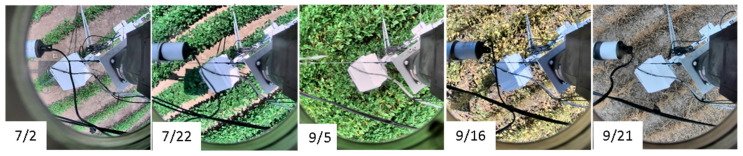
Selected RGB photos captured at one of the 12 positions (−146 degrees) around the EcoSpec tower during the 2015 growing season. These photos show phenology of plants and provide contextual information to aid analyses of measurements collected using the EcoSpec system.

RGB photos on the selected dates during the 2015 growing season that were captured using the EcoSpec system are shown in Figure 4. Soybean plants planted in mid-April 2015 covered approximately 25% of the ground surface in the beginning of July. Foliage cover exceeded 60% of the ground in two weeks (Figure 4) and completely closed by early August. The plants started to show a sign of senescence in early September and completely senesced by mid-September. RGB photos of ground surface documents plant conditions (e.g., stress, disease) and phenology and would provide valuable information for interpreting optical and infrared measurements. For example, certain patterns in spectral reflectance values or signatures may be evaluated based on plant or land surface conditions captured in the photos. Causes of sudden changes in spectral reflectance and/or vegetation indices may be identified using the photos. The RGB photos may also be used to investigate the effects of soil exposure, leaf orientation, and shadow on optical and infrared measurements.

**Figure 5:**
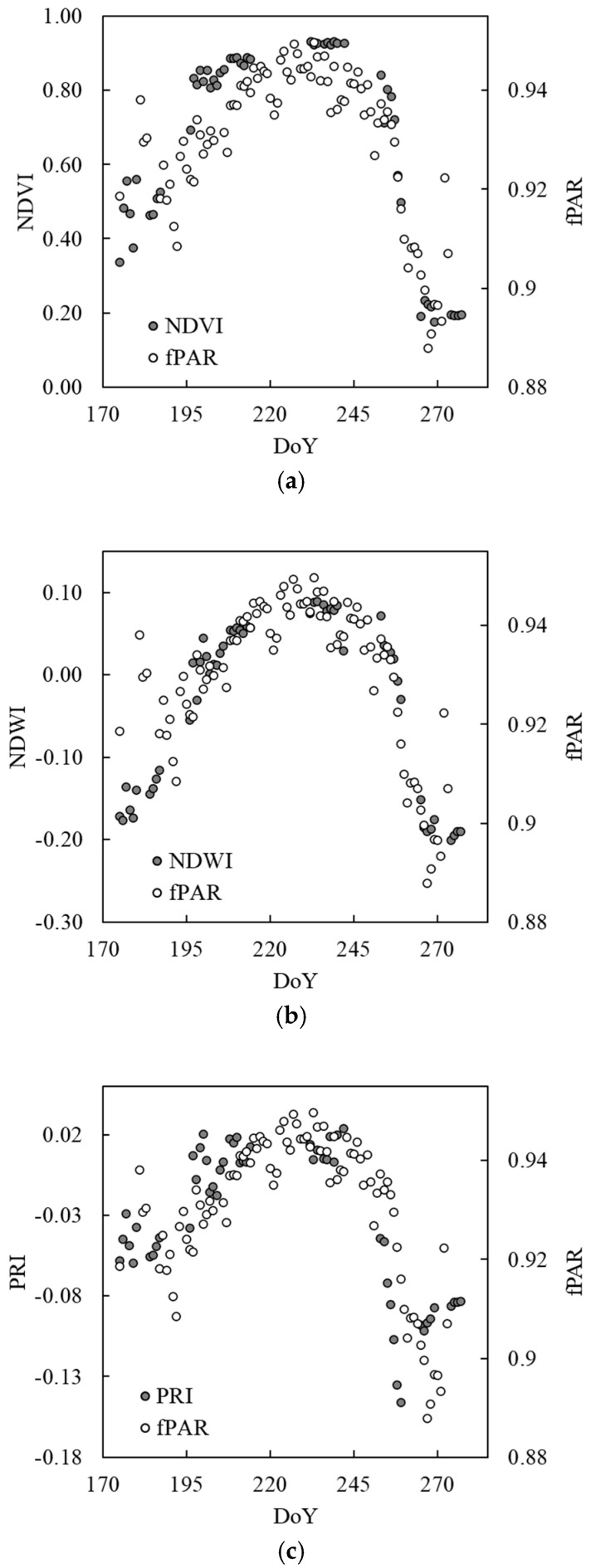
Seasonal trajectories of (**a**) NDVI, (**b**) NDWI, and (**c**) PRI compared with the fraction of photochemically active radiation absorbed by land surface (fPAR) of the 2015 growing season that was measured using the eddy covariance system.

Seasonal trajectories of the NDVI, NDWI, and PRI along with the trajectory of the fPAR that was measured using the eddy covariance system are shown in Figure 5. All three indices increased toward the peak growing season of late July to early August and decreased toward the end of the season. These patterns correspond to that of fPAR throughout the growing season (Figure 5). Because fPAR is a biophysical variable known to directly relate with the primary productivity or photosynthesis [35,36,37], this comparison between spectral vegetation indices and fPAR indicates the potential of spectral reflectance measurements of land surface collected at a high temporal frequency throughout the growing season to investigate ecosystem functions, such as photosynthesis and respiration.

**Figure 6:**
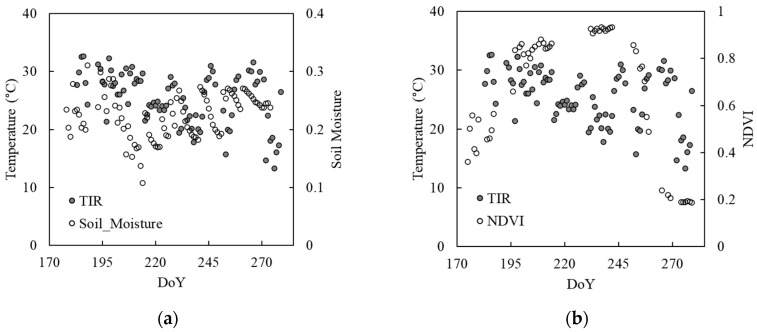
Seasonal trajectories of TIR temperature with (**a**) soil moisture and (**b**) NDVI of the 2015 growing season.

Seasonal trajectories of TIR temperature, soil moisture, and NDVI of the 2015 growing season are shown in Figure 6. The TIR values indicating plant canopy temperature appear to show a pattern in relation to soil moisture in early August (DoY 213) (Figure 6a), when plants matured as indicated by NDVI (Figure 6b). High TIR values correspond to low soil moisture during the peak growing season. The relationship dissipates toward the end of the season. This suggests the benefit of simultaneous measurement of TIR with optical and near/shortwave infrared reflectance for studying plant responses to environmental shifts and variability, particularly during the peak growing season.

## 4. Conclusions

Understanding interactions of ecosystem functions and climate across varying spatial and temporal scales could improve climate forecasting capability. However, determining the linkage between very different scales (e.g., global and local) is particularly difficult because key processes at each scale operate at different rates and create complex heterogeneity and dynamics. Recognizing that existing instrumentation would not fully support the type of data collection needed to investigate the interaction of two extreme scales, we developed a tower-based remote sensing system to automatically collect a range of optical and infrared measurements of land surface across the visible to TIR spectral region throughout the day over the growing season. While many existing near-surface remote sensing systems continuously collect measurements in an automated manner over an extended period of time, the uniqueness of the EcoSpec system includes the following:

1. Collection of measurements from 12 locations spanning a 300 degree rotation of the platform to account for heterogeneity of the land surface.
2. Integration of five optical and infrared sensors to collect a suite of measurements that complements hyperspectral reflectance measurements, enabling various modeling approaches, such as surface and sky temperature, direct and diffused sunlight, incoming and outgoing albedo, and RGB photos of the land surface.
3. Custom software to control synchronized movements and functions of the system components so that every sensor collects measurements properly without interference of other sensors. This software manages a specific order or timing of actuator movement, reference reflectance measurement, target reflectance measurement, and platform movement in a compact package.
4. Portability of the system as being powered by a relatively compact power generating system, such as the solar power system evaluated during the entire testing period, which allows for deployment at a range of sites, including existing research stations/observatories, short-term/temporary locations, remote locations, and agricultural fields.

When examining the measurements collected using the system, temporal trajectories between narrow-band spectral vegetation indices—NDVI, NDWI, and PRI—and a biophysical variable directly correlated with the primary production—fPAR—were strongly correlated. TIR temperature that correlates with the MSI at the diurnal scale appeared to correspond to soil moisture across the season. These patterns suggest that hyperspectral reflectance and simultaneously collected complementary optical and infrared measurements could provide valuable observations for investigating ecosystem functions, such as photosynthesis or gross primary production and possibly ecosystem respiration in response to environmental changes, which would provide critical information to understand interactions between biosphere and atmosphere at its interfaces.

Optical and infrared measurements of land surface—plants and surface soils—properties would provide critical data for understanding ecosystem functions and their responses and contributions to environmental changes and variability. Instrumentation that allows measurements from multiple locations at high temporal frequency would enable experiments and modeling with great flexibility by providing replicates of samples and options for temporal aggregation. This type of near-surface remote sensing system can be used to complement existing science observatories to validate satellite remote sensing observations, link local- and regional/global-scale phenomena, and ultimately improve our ability to forecast ecosystem functions and climate. When applying this system for precision agriculture, measurements would be useful for studying crop growth, detecting disease and pests, and comparing traits of breeds or cultivars to improve our future food security.

The EcoSpec system developed in this study is best suited for research that aims to explore various modeling and analysis approaches to understand fine-scale plant properties and/or ecosystem functions for heterogeneous land. When studying a relatively uniform and homogenous area, an instrument that continuously collects measurements from a single patch of land may be sufficient. When the types of measurements required for research are already known, the system having only those sensors necessary may be more efficient than using a highly equipped system like the EcoSpec system.

The examples presented in this paper utilized a preliminary method to account for BRDF and did not consider the effects of varying sky conditions on hyperspectral measurements. When more sophisticated models are applied for data cleansing, the quantity and quality of the hyperspectral reflectance measurements useful for the analysis are expected to improve significantly.

## Figures and Tables

**Table 1:** EcoSpec System Sensors and Components.

Instrument (Model, Manufacture)	Description
Spectrometer (ASDFieldSpec4 Std-Res, Malvern Panalytical; Cambridge, UK)	Spectral reflectance of land surface using 2150 spectral channels ranging from 350 to 2500 nm. Instrument with a 1.5 m fiber optic cable having a 25 degree field of view (FOV). Height of installation: 3–7 m above ground. Variables: Spectral reflectance (350–2500 nm) and spectral vegetation indices. ^1^ Frequency of measurement: ~1 min ^2^.
Thermal IR sensor (SI-111, Apogee Instruments Inc.; Logan, KY, USA)	Radiant temperature of canopy and ground surface. Height of installation: 3–7 m above ground. Variables: Surface temperature (plants and exposed soil) and sky temperature. Frequency of measurement: 1 min.
Red-green-blue (RGB) camera (Q1604, Axis; Lund, Sweden)	Plant conditions; visual and contextual information within the field of view of the spectrometer and its surroundings. Height of installation: 3–7 m above ground. Variable: Contexture information of land surface, such as plant conditions and land cover composition and structure. Frequency of measurement: 1 min.
Diffuse radiometer (RSR2, Irradiance Inc.; Cambridge, MA, USA)	Components of incoming light, such as direct and diffused light (2–6 m above ground). Variables: Total incoming light, direct light, sky light, sky temperature, and air temperature. Frequency of measurement: 1 min.
Albedometer (Dual-Pyranometer 8104, Schenk; Grass Valley, CA, USA)	Albedo of incoming and outgoing light. Height of installation: 2–6 m above ground. Variables: Downwelling irradiance and upwelling irradiance. Frequency of measurement: 1 min.
Pan-tilt unit (PTU-D300, FLIR; Wilsonville, AL, USA)	Rotating platform for the upper package, including spectrometer, thermal IR sensor, and RGB camera, in order to collect measurements at 12 positions around the tower (300°). Payloads: 70 lbs.
Enclosure (ENC 14/16-MM-NC, Campbell Scientific Inc.; Logan, KY, USA)	House spectrometer and RGB camera.
Actuator (PA-15-8-11, Progressive Automations; Arlington, TX, USA)	Extend and retract the white reference panel to enable white reference measurement right before each measurement from land surface.
White reference panel (Spectraron, Labsphere; North Sutton, NH, USA)	Provide a reference surface for white reflectance calibration. The highly reflective surface has 99% reflectivity across the spectral range.
Single-board computers (Raspberry Pi)	Provide commands to all system components and temporary data storage.
DC/AC converter	Convert DC power from the solar power system to AC to power the instruments.
Enclosure (Campbell Scientific Inc.; Logan, KY, USA)	House single-board computer, CR1000 data logger, and cellular modem.

^1^ Reflectance values are transformed to 100+ spectral vegetation indices that are known to correlate with plant and ecosystem properties such as biomass, plant greenness or pigment, structure, and moisture including Normalized Difference Vegetation index (NDVI), Normalized Difference Water Index (NDWI), Photochemical Reflectance Index (PRI), Enhanced Vegetation Index (EVI), Chlorophyll Index (CI), Moisture Stress Index (MSI), Water Band Index (WBI), and Modified Chlorophyll Absorption in Reflectance Index (MCARI). ^2^ Depending on the level of irradiance.

## Supplementary Materials

The following are available online at https://www.mdpi.com/1424-8220/20/19/5463/s1, Video S1: Movement of the EcoSpec system and data collection in the agricultural field.
